# Analysis of the Human Proteome in Subcutaneous and Visceral Fat Depots in Diabetic and Non-diabetic Patients with Morbid Obesity

**DOI:** 10.4172/jpb.1000361

**Published:** 2015-06-08

**Authors:** Lingling Fang, Kyoko Kojima, Lihua Zhou, David K Crossman, James A Mobley, Jayleen Grams

**Affiliations:** 1 Ningbo Lihuili Hospital; Ningbo, Zhejiang, China; 2 Department of Surgery, University of Alabama at Birmingham; Birmingham, AL, USA; 3 Comprehensive Cancer Center, University of Alabama at Birmingham; Birmingham, AL, USA; 4 Heflin Center for Genomic Science, University of Alabama at Birmingham; Birmingham, AL, USA; 5 Department of Genetics, University of Alabama at Birmingham; Birmingham, AL, USA; 6 Department of Surgery, Birmingham Veterans Administration Medical Center, Birmingham, AL, USA

**Keywords:** Adipose tissue, Diabetes, Obesity, Proteome

## Abstract

No longer regarded as simply a storage depot, fat is a dynamic organ acting locally and systemically to modulate energy homeostasis, glucose sensitivity, insulin resistance, and inflammatory pathways. Here, mass spectrometry was used to survey the proteome of patient matched subcutaneous fat and visceral fat in 20 diabetic vs 22 nondiabetic patients with morbid obesity. A similar number of proteins (~600) were identified in each tissue type. When stratified by diabetic status, 19 and 41 proteins were found to be differentially abundant in subcutaneous fat and omentum, respectively. These proteins represent pathways known to be involved in metabolism. Five of these proteins were differentially abundant in both fat depots: moesin, 78 kDa glucose-regulated protein, protein cordon-bleu, zinc finger protein 611, and cytochrome c oxidase subunit 6B1. Three proteins, decorin, cytochrome c oxidase subunit 6B1, and 78 kDa glucose-regulated protein, were further tested for validation by western blot analysis. Investigation of the proteins reported here is expected to expand on the current knowledge of adipose tissue driven biochemistry in diabetes and obesity, with the ultimate goal of identifying clinical targets for the development of novel therapeutic interventions in the treatment of type 2 diabetes mellitus. To our knowledge, this study is the first to survey the global proteome derived from each subcutaneous and visceral adipose tissue obtained from the same patient in the clinical setting of morbid obesity, with and without diabetes. It is also the largest study of diabetic vs nondiabetic patients with 42 patients surveyed.

## Introduction

Having an overall prevalence of 34.9% among US adults in 2011–2012, obesity is an epidemic that has tremendous medical and socioeconomic impact [1]. With the increasing prevalence of obesity, there has been an increase in the prevalence of type 2 diabetes and as many as 23% of patients with morbid obesity have diabetes [2,3]. Given the association of type 2 diabetes with obesity, adipose tissue has become a target of investigation in understanding the pathogenesis of this disease. No longer regarded as simply a storage depot, fat is a dynamic organ secreting adipocytokines that act locally and systemically to modulate energy homeostasis, insulin resistance, and an inflammatory state [4,5]. Further, the specific contribution of adipose tissue is depot dependent, and visceral fat is thought to play a greater role in dysmetabolism than subcutaneous fat [6–11]. It is worth noting, however, that the majority of patients with obesity do not have frank diabetes.

In order to obtain a more global understanding of adipose tissue, various “–omics” strategies have been performed and include comprehensive gene, lipid, metabolite, and proteomic analysis. There has been particular interest in proteomics, since there is a variable correlation between gene and protein expression [12]. Previous studies have investigated the secretome [13–15], organellar proteome [16], as well as isolated adipocyte and whole adipose tissue proteome [17–24]. Of these reports, eight have focused on the global proteome derived from whole adipose tissue in humans. These include various fat depots, such as subcutaneous fat [17,19,20], visceral fat [22–24], and both subcutaneous and visceral fat depots [18–21]. Regarding clinical states, Boden et al. examined the proteome from subcutaneous fat in patients with obesity and insulin resistance vs lean and insulin sensitive [17], Insenser et al. from both adipose tissue depots in patients with obesity [21], Oliva et al. in visceral fat from women who had gestational diabetes [23], and Murri et al. and Kim et al. in visceral fat from lean patients with type 2 diabetes mellitus [22–24]. To our knowledge, our study is the first to survey the global proteome derived from each subcutaneous and visceral adipose tissue obtained from the same patient in the clinical setting of morbid obesity, with and without diabetes. It is also the largest study of diabetic vs nondiabetic patients with 42 patients surveyed vs 24 patients in the Oliva et al. study on gestational diabetes and 16 patients in the study by Murri et al., the largest study published to date on type 2 diabetes [22,23]. Here, we have specifically focused on proteins that are differentially abundant in diabetic vs nondiabetic patients from each tissue type, while also highlighting a few proteins that were similar across both tissue types.

## Materials and Methods

### Biological samples

The study was approved by the Institutional Review Board at the University of Alabama at Birmingham, and written informed consent was obtained from all participants. In total, 42 patients with morbid obesity were recruited into the study from June 21, 2005 to June 11, 2008. There were 22 nondiabetic and 20 diabetic female patients. All patients underwent routine medical screening for preoperative evaluation and had been approved for bariatric surgery. Data collected included patient demographics (age, sex), anthropomorphic measurements (height, weight, BMI), obesity-associated medical problems (type 2 diabetes mellitus, hypertension, hyperlipidemia), and fasting laboratory evaluation (fasting glucose and lipid levels). Clinical diagnoses were based on active medical therapy for a known medical condition or established practice guidelines [25]. Adipose tissue was obtained at the time of bariatric surgery, specifically Roux-en-Y gastric bypass, from two sites: abdominal subcutaneous fat and omentum. Samples were immediately placed in DMEM (Gibco, Carlsbad, CA, USA) and transported to the laboratory, where they were frozen in liquid nitrogen and stored at −80°C.

### Sample preparation and data acquisition

Each fat depot specimen from each patient was prepared and analyzed independently without pooling. In order to improve proteomic coverage, tissues were first delipidated following the Bligh and Dyer method, and the protein-containing aqueous and middle layers and any remaining tissue remnants were transferred to a fresh eppendorf tube [26]. Proteins were further extracted using tissue protein extraction reagent (T-PER, Thermo Scientific, San Jose, CA) per manufacturer’s instructions. All protein extracts were depleted of albumin using ProteaPrep albumin depletion spin columns (Protea Biosciences, Morgantown, WV) following manufacturer’s instructions. The flow-through fractions obtained after the immunodepletion step were collected, exchanged into 100 mM ammonium-bicarbonate (AmBic), and concentrated to approximately 20–30 µL using Amicon Ultra 3 kDa MWCO centrifugal filter devices (Millipore, Billerica, MA). Protein concentrations were determined with the BCA protein assay (Pierce, Thermo Scientific). For each sample, up to 5 µg of protein was concentrated to near completion using a Savant SpeedVac Concentrator (Thermo Fisher Scientific), solubilized, and denatured in 5 µL of 6 M urea, pH 8.0. The samples were then reduced with 10 mM DTT for 60 min, alkylated with 40 mM iodoacetamide (IAA) for 45 min in the dark, followed by quenching the IAA with 40 mM DTT for 15 min, all at room temperature. The sample was then diluted 1:10 using 1 mM CaCl_2_ in 50 mM Tris-HCl, and enzymatically digested with Trypsin Gold (Promega, Madison, WI) overnight according to manufacturer’s instructions, followed by acidification to pH 3–4 with 10% formic acid, with a final concentration of ~100 ng/µL for analysis by liquid chromatography-mass spectrometry (LC-MS). In order to measure run-to-run variation, we chose a random omental sample of high abundance to use as an internal quality control, which was analyzed between every 8–10 specimens (n=4–5 for each tissue type, n=9 for the entire set).

For each sample, 1 µg of peptide digest were analyzed using a linear trap quadropole XL (LTQ XL) ion trap mass spectrometer equipped with a nano-electrospray source, and a Surveyor Plus binary high-pressure liquid chromatography (HPLC) pump (Thermo Scientific, San Jose CA) using a split flow configuration. While most specimens were run in duplicate, due to limited protein acquired for a number of the specimens in this study, duplicate injections could not be carried out for all samples. Therefore, it is important to note that all downstream informatics analyses were carried out on single-injection data in order to avoid the potential of skewing results with single vs duplicate LCMS runs. Separations were carried out using a 150 µm × 13 cm pulled tip C-18 column (Jupiter C-18 300 A, 5 micron, Phenomenex, Torrance, CA). The HPLC was set up with two mobile phases that included solvent A (0.1% FA in ddH_2_O), and solvent B (0.1% FA in 85% ddH_2_O/15% ACN), and was programmed as follows: 15 min at 0% B (2 µL/min, load and desalt), 100 min at 0%–50% B (~0.5 nL/min, analyze), 20 min at 0% B (2 µL/min, equilibrate). During the first 15 minutes of loading and desalting, the source was set at 0.0 volts. The LTQ XL was operated in data dependent triple play mode, with a survey scan range of 300–1200 *m/z*, followed by an ultra-zoom scan used for charge state determination (~20k resolution 400 *m/z*) and an MS2 scan, both carried out with 2.0 Da isolation widths on the 3 top most intense ions. MS data were collected in profile mode for all scan types. Charge state screening and dynamic exclusion were enabled with a minimum signal intensity of 2000, a repeat count of 2, and exclusion duration of 90 s for ions +/− 1.5 *m/z* of the parent ion. The automatic gain control settings were 3×104, 5×103, and 1×104 ions for survey, zoom, and CID modes respectively. Scan times were set at 25, 50, and 100 ms for survey, zoom, and collision-induced dissociation (CID) modes, respectively. For CID, the activation time, activation Q, and normalized collision energy were set at 30 ms, 0.25, and 35% respectively. The spray voltage was set at 1.9 kV following the first 15 minutes of loading, with a capillary temperature of 170°C.

### Data analysis

The XCalibur RAW files were centroided and converted to MzXML and the mgf files were then created using both ReAdW and MzXML2Search, respectively (http://sourceforge.net/projects/sashimi/). The data was searched using SEQUEST (v27 rev12, .dta files), set for two missed cleavages, a precursor mass window of 0.45 Da, tryptic enzyme, variable modification M at 15.9949, and static modifications C at 57.0293. Searches were performed with a human subset of the UniRef100 database (Human; extracted January 2014, virus entries excluded; 29,171 entries), which included common contaminants such as digestion enzymes and human keratins.

Identified peptides were filtered, grouped, and quantified using ProteoIQ v2.3.04 (Premierbiosoft, Palo Alto, CA). Only peptides with charge state of ≥ 2+ and a minimum peptide length of 6 amino acids were accepted for analysis. ProteoIQ incorporates the two most common methods for statistical validation of large proteome datasets, false discovery rate (FDR), and protein probability [27–29]. Relative quantification was performed via spectral counting [30,31], and spectral count abundances were normalized between samples [32]. The FDR was set at <1% cut-off, with a total group probability of ≥ 0.7 and peptides ≥ 2 assigned per protein.

### Statistical analysis

Three filters were used to determine significance, 1) commonality, where the protein of interest had to be observed in greater than 50% of any one group +/− diabetes, 2) Wilcoxan with a filter cut off of ≤ 0.05, and 3) relative protein abundance ratios as determined with normalized spectral counting, set at ≥ 1.5 fold change. The fold change was determined empirically by analyzing the inner-quartile data from a control group experiment (omental fat depot/non-diabetes) using ln-ln plots, where Pearson’s correlation coefficient (R) was 0.98, and >99% of the normalized intensities fell between +/−1.5 fold (data not shown). In each case, all three tests (commonality, Wilcoxon, and fold change) had to pass. For the measurement of intra-variation, ln-ln plots were generated from the normalized spectral count (N-SC) data, and the Pearson’s correlation coefficient (R) was calculated.

### Systems biology analysis

Those proteins which were found to have significantly changed between any two groups were further filtered for biological significance, and also as a means of pseudo-validation by comparing key biological functions to metabolic related pathways. The systems biology analyses are carried out with Gene Ontology (Gene Ontology Consortium, http://www.geneontology.org/) [33] and Ingenuity Pathway Analysis (IPA) (Ingenuity Systems, Redwood City, CA, http://www.ingenuity.com). First, all identified proteins in subcutaneous and omental fat were uploaded into Gene Ontology and IPA. Second, proteins that had differential abundance in subcutaneous fat or omentum from diabetic vs nondiabetic patients were uploaded into Gene Ontology and IPA for analysis. For canonical pathways, no expression value cutoff was selected and both upregulated and downregulated fold change proteins were included. The significance of the association between the data set and the canonical pathway was measured in 2 ways: 1) ratio of the number of molecules from the data set mapping to the pathway and the total number of molecules that map to the canonical pathway and 2) Fisher’s exact test to calculate a p-value with p<0.05 set as the threshold value. The comprehensive Ingenuity knowledge base was also searched for biological processes of interest: actin cytoskeleton, lipid metabolism, oxidative stress, cell differentiation, cell signaling, mitochondrial function, and ER stress (Supplemental Tables 2–5).

### Western blot analysis

Protein was isolated from the subcutaneous and visceral adipose depots of six additional patients, three nondiabetic and three diabetic, who were not included in the initial specimens from which the proteomic data were generated. Briefly, adipose tissue was homogenized on ice in cold modified Margolis lysis buffer: 50 mM HEPES, pH 7.5, 150 mM NaCl, 1.5 mM MgCl_2_, 1 mM EGTA, 200 µM sodium orthovandatae, 10 mM sodium pyrophosphate, 100 mM sodium fluoride, 1% TritonX-100 with protease inhibitors (1 mM PMSF, 100 µM leupeptin, 100 µM aprotinin). All reagents were purchased from Sigma Aldrich (St. Louis, MO). Protein was isolated by centrifugation at 14,000 × g at 4°C three times to remove the lipid. Protein concentration was determined by the Thermo Scientific Pierce BCA Protein Assay Kit (Rockford, IL) and 30 µg were used for separation by SDS-PAGE, followed by transfer to Immobilon-PSQ PVDF membrane (Millipore, Billerica, MA). Membranes were blocked for 1–2 h with 5% nonfat dry milk at room temperature, followed by primary antibody incubation overnight at 4°C, and secondary antibody incubation for 1 h at room temperature. Decorin, cytochrome c oxidase subunit 6B1, and 78 kDa glucose-regulated protein (GRP78) primary antibodies were purchased from Abcam, Inc (Cambridge, MA) and used at 1:1000 dilution. Bands were visualized by chemiluminescence (Thermo Scientific, Rockford, IL) and quantitated using ImageJ version 1.47 (National Institutes of Health, Bethesda, USA). Protein loading was normalized to GAPDH.

## Results and Discussion

### Patient data

In total, 42 patients consented to the study and underwent matched adipose tissue biopsies of subcutaneous fat and omentum, 20 patients had type 2 diabetes mellitus while 22 did not. Of the diabetic patients, data regarding the duration of diabetes was available for 14 patients with a mean of 6.4 ± 5.63 years and median of 5.5 years (IQR=3–8). Eleven patients were on single agent therapy with metformin in the biguanide class, and one patient was on insulin therapy alone. The remaining 8 patients were on combination therapy including a biguanide (7), thiazolidinedione (6), sulfonylurea (4), insulin (2), or a dipeptidyl peptidase-4 inhibitor (1). As expected since the subjects were stratified based on diabetes status, there was a significant difference between the two groups in fasting glucose (p=0.035) (Table 1).

The diabetic patients also had a significantly higher incidence of hyperlipidemia (45.0 vs 13.6%, respectively; p=0.025) (Table 1). All 9 of the diabetic patients with hyperlipidemia were on medication: seven patients were on statin therapy alone; one patient was on niacin and fenofibrate (a peroxisome proliferator receptor alpha agonist); and one patient was on a bile acid sequestrant alone. Of the nondiabetic patients, two of the three patients were on medical therapy: one patient on statin therapy with a cholesterol absorption inhibitor and the other on statin therapy alone. There was no significant difference in serum lipid levels between the two groups.

### Subcutaneous vs visceral fat

**Figure 1:**
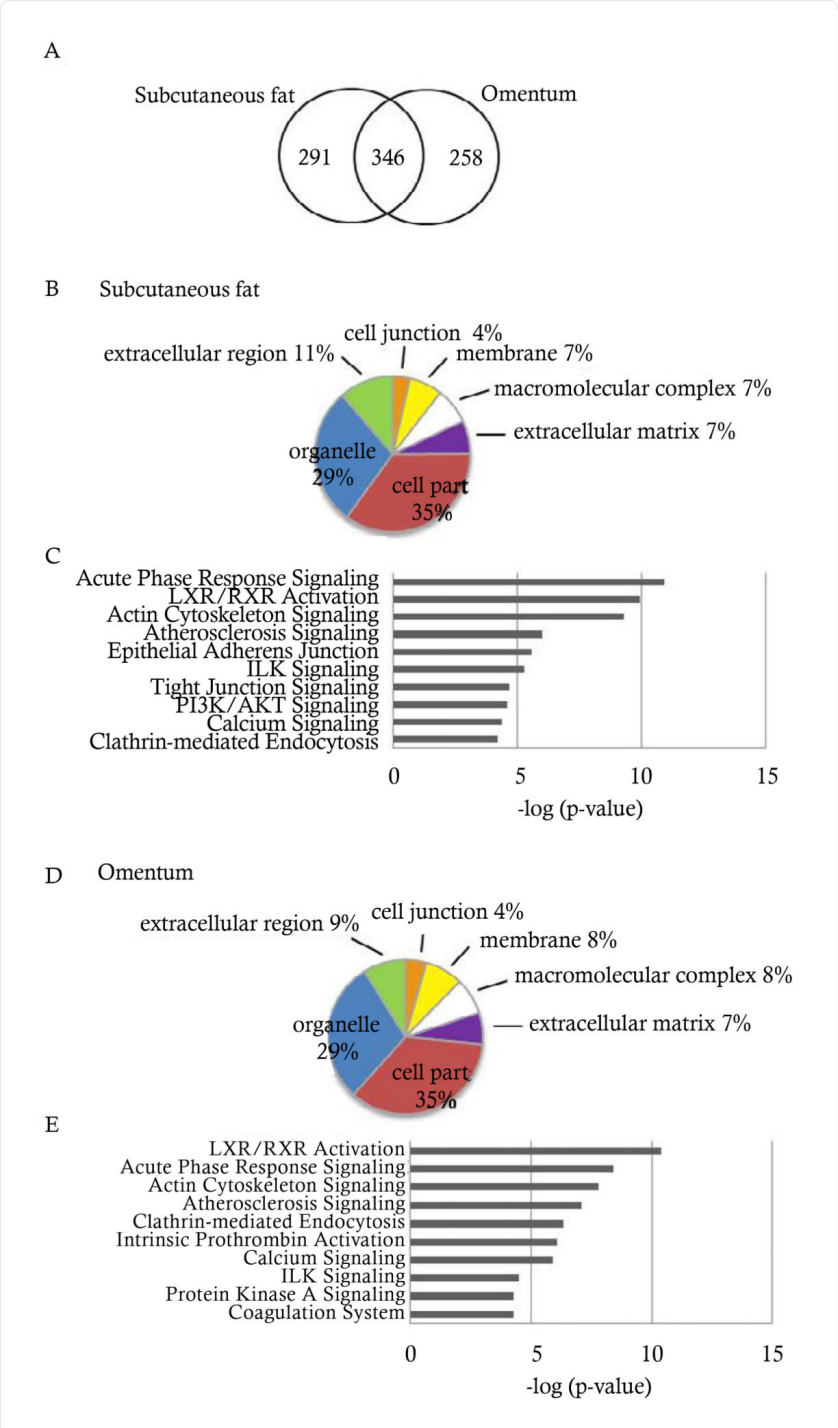
Comprehensive proteome of subcutaneous and omental adipose tissue. (A) Venn diagram depicting the number of proteins identified in subcutaneous fat and omentum by proteomic analysis. (B,D) Subcellular compartment distribution in subcutaneous fat and omentum, respectively. (C,E) Top canonical pathways in subcutaneous fat and omentum, respectively.

In order to survey the proteomes for each of the subcutaneous and omental tissues, a straightforward workflow comprising delipidation and immune-depletion was applied. Due to the high level of albumin and lipid contamination, in many cases, it was difficult to extract up to 1–2 µg of clean protein; however, the depletion steps followed by desalting and concentrating with 3 kDa cut-off columns, resulted in very clean tryptic peptides for LC-ESI-MS2 analysis. Once analyzed, peptides were filtered, grouped to their corresponding proteins, and quantified using ProteoIQ v2.03.04. Only peptides identified in a human subset of the UniRef100 database with minimal peptide length of 6 amino acids and ≥ 2 peptides assigned per protein were included. Using this approach, 637 proteins were identified in subcutaneous fat and 604 proteins in omentum. A detailed list of these proteins has been provided in Supplemental Table 1. Of these, 346 proteins were found in both fat depots (Figure 1A). Cellular components were examined using Gene Ontology (Figure 1B and D). The distribution of proteins into the cellular components categories is essentially identical between the two depots, subcutaneous fat and omentum, with cell part (35%), organelle (29%), and extracellular region (11 vs 9%, respectively) comprising the top three categories. Proteins were further analyzed according to their canonical pathways by IPA (Figure 1C and E). The top three pathways were identical in both adipose tissue depots: acute phase response signaling, LXR/RXR activation, and actin cytoskeleton signaling. Other pathways were also shared (atherosclerosis signaling, ILK signaling, calcium signaling, and clathrin-mediated endocytosis), while some were unique to fat depot.

### Diabetes vs non-diabetes

**Figure 2:**
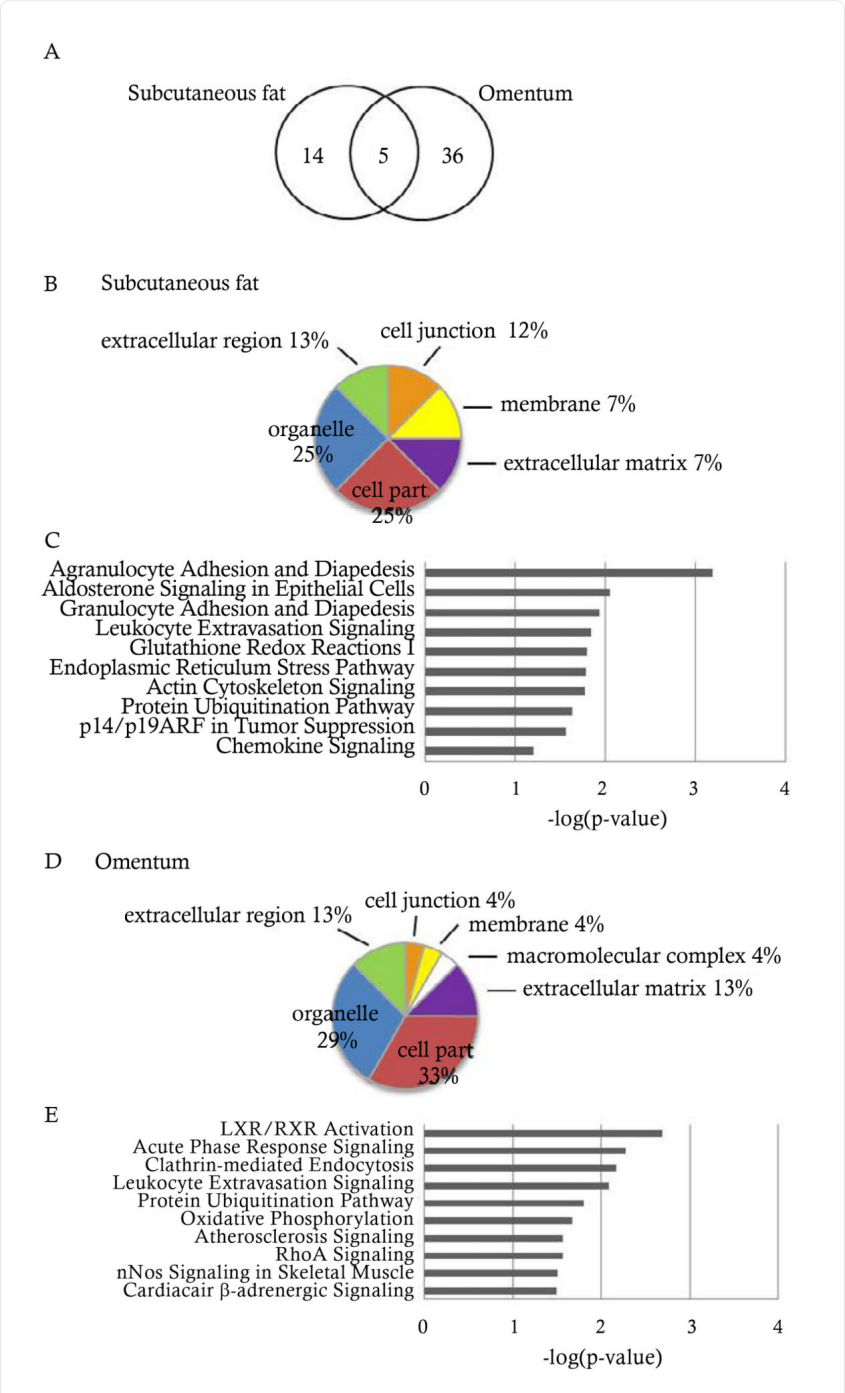
Comprehensive proteome of proteins that are differentially expressed in diabetic vs non-diabetic patients in subcutaneous fat and omentum. (A) Venn diagram depicting the number of proteins differentially expressed in diabetic vs nondiabetic patients in subcutaneous fat and omentum. (B,D) Subcellular compartment distribution in subcutaneous fat and omentum, respectively. (C,E) Top canonical pathways in subcutaneous fat and omentum, respectively.

To determine putative proteins involved in the pathophysiology of diabetes in patients with obesity, patients were analyzed according to diabetes status and proteome profiles of each fat depot were compared. Proteins were filtered based on at least a 1.5 fold change, p value <0.05, and 50% commonality cutoff within the dataset. In total, 19 proteins were differentially expressed in subcutaneous fat and 41 proteins in omentum (Figure 2A and Tables 2, 3). Five proteins were found to be differentially abundant in both fat depots and showed a similar up- or down-regulation: moesin, GRP78, protein cordon-bleu, zinc finger protein 611, and cytochrome c oxidase subunit 6B1.

The cellular components of the differentially abundant proteins in subcutaneous fat and omentum were determined. Again, the top cellular components were shared and included cell part, organelle, and extracellular region (Figure 2B, D). However, canonical pathway analysis revealed more significant differences between the two fat depots, with only two pathways in common: leukocyte extravasation signaling and protein ubiquitination (Figure 2C, E). Interestingly, the differentially abundant proteins in the omentum better represented pathways known to contribute to cellular energy homeostasis and/or the pathogenesis of type 2 diabetes mellitus: LXR/RXR activation, acute phase response signaling, clathrin-mediated endocytosis, leukocyte extravasation signaling, and oxidative phosphorylation.

Because type 2 diabetes is characterized by inflammation and dysmetabolism, the proteins were also grouped according to the following biological and metabolic processes: actin cytoskeleton, lipid metabolism, oxidative stress, cell signaling, mitochondrial function, and ER stress (Table 4). The actin cytoskeleton group comprised the largest number of proteins that were differentially abundant according to diabetic status. This is consistent with other published proteome studies as well as with the previously described role of the actin network in insulin signaling and GLUT4 translocation to the cell membrane [22,23,34,35]. In our study, most of the proteins were decreased, potentially consistent with the finding that chronic low treatment with insulin results in decreased GLUT4 translocation and glucose uptake in the murine 3T3-L1 adipocyte cell line, and a marked loss of cortical filamentous actin [35]. Further, in our proteomic analysis, unconventional myosin 1c abundance was 6.36-fold decreased and girdin 4.85-fold decreased in the omentum of diabetic vs nondiabetic patients. Myosin 1c has been shown to contribute to the translocation of GLUT4-containing vesicles to the plasma membrane in adipocytes [36,37]. Previous studies have demonstrated a correlation between girdin expression with insulin sensitivity in human myotubes and overexpression to increase insulin sensitivity in murine myoblasts [38].

Three proteins of interest were selected for verification by western blot analysis based on criteria that included potential biological function in diabetes or metabolism, degree of fold-change or similarity in change between both tissue types, and statistical significance. The validation study consisted of proteins isolated from six patients, three nondiabetic and three diabetic, who were independent of the initial 42 patients used to determine the proteome. In contrast to the patients used to generate the dataset, none of the six patients used in the validation had hyperlipidemia and none were on lipid-lowering agents. Of the diabetic patients, all three were on single agent therapy with metformin. Importantly, no specific effect of metformin on adipocyte function has been reported to date.

**Figure 3:**
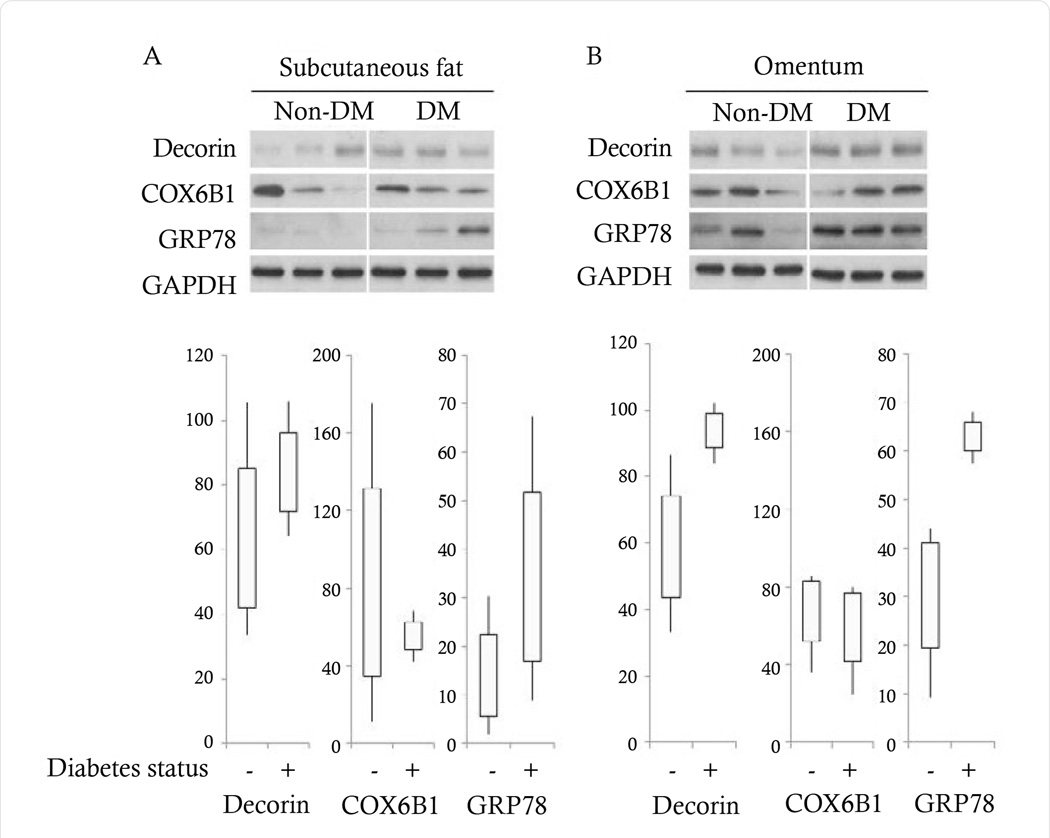
Validation of select proteins identified by proteomic analysis to have differential expression in diabetic vs nondiabetic patients. (A,B) Western blot analysis of decorin, cytochrome c oxidase subunit 6B1 (COX6B1), and 78 kDa glucose-regulated protein (GRP78) in nondiabetic and diabetic patients in subcutaneous fat vs omentum, respectively.

As observed in Figure 3, both decorin and GRP78 were markedly increased in diabetic patients vs nondiabetic patients in the omentum, as observed in the MS analysis. Subcutaneous fat also showed increases in both of these proteins, but the differences were not as distinct as they were for omentum, also consistent with the proteomic results. Although there was a trend for increased GRP78 in the omentum of diabetic patients (p=0.06), there was no statistically significant differences in any of the comparisons based on diabetic status. This is likely due to marked individual variability among patients as well as to the small sample size based on tissue availability. This study does implicate both of these proteins in obesity and T2DM, however, and both in vitro and in vivo experiments are necessary to further investigate the contribution of these two proteins to dysmetabolism. Importantly, we are encouraged that our results are consistent with previous work demonstrating, 1) decorin gene expression is higher in visceral vs subcutaneous adipose tissue, 2) circulating plasma decorin is increased in diabetic patients, and 3) plasma decorin levels are associated with waist-to-hip ratio or central adiposity in humans [39]. An N-terminal truncation of decorin (ΔDecorin) localizes to the surface of adipose progenitor cells in mice and has been proposed to be a putative resistin receptor [40]. To our knowledge, no studies in humans have discriminated between the full length or truncated proteoforms. Here, a total of 10 peptides mapped to decorin with the N-terminal-most peptide identified starting at aspartic acid 45 in humans vs leucine 45 in mice (this leucine is at position 50 in humans). Similar to the mouse truncation, if this aspartic acid 45 were the start of the protein in human adipose tissue, this proteoform would also lack the SerGly that serves as the glycanation site. However, our results do not exclude that the full-length mature core protein was not present, only that a peptide was not identified that mapped to this region of decorin. Expression of the mouse ΔDecorin vs full-length decorin in the 3T3-L1 adipocyte cell line resulted in an increased proliferation of cells, and inhibition of large lipid droplets, consistent with reports in humans that there is an increase in the fraction of small adipocytes with insulin resistance and type 2 diabetes [41,42].

GRP78 is known to play a key role in the ER stress pathway. Under normal conditions, it is associated with the luminal domain of proteins involved in the unfolded protein response (UPR) signaling pathways: PKR-like endoplasmic reticulum kinase (PERK), inositol-requiring enzyme 1 (IRE1), and activating transcription factor 6 (ATF6). Under conditions of ER stress, accumulated unfolded or misfolded proteins in the ER lead to the recruitment of GRP78 away from the luminal domains of these signaling molecules, allowing their activation. Early activation of UPR signaling helps to restore the protein folding capacity of ER. However, unresolved ER stress leads to prolonged activation of UPR signaling which is an underlying mechanism of inflammation and metabolic diseases including obesity and diabetes [43–45].

Differences in protein abundance of cytochrome c oxidase subunit 6B1 (Cox6B1) in diabetics vs nondiabetics was not validated by western blot. Cox6B1 is one of the polypeptides comprising mitochondrial Complex IV. Data suggests that Complex IV gene expression and activity may be downregulated in diabetes, but any specific role for Cox6B1 has yet to be elucidated [46,47]. Again, lack of a statistically significant difference could be due to marked individual variability among patients as well as to the small sample size based on tissue availability as noted above. However, in contrast to the findings with decorin and GRP78, there was no trend toward differential abundance in the nondiabetic and diabetic patient groups and in fact the two groups had marked overlap in Cox6B1 abundance. Thus, we think it is more likely that cytochrome c oxidase subunit 6B1 is not differentially abundant based on diabetic status and may reflect the differences in the patient population used for determining the proteome vs validation. For example, the initial specimens used to generate the proteome data were taken from patients who also had a significantly higher incidence of hyperlipidemia and were being treated for it.

We also compared our results to the four previously published studies investigating the proteome based on insulin resistance or the presence of type 2 diabetes (Table 5) [17,22–24]. Of these four studies, only the study by Boden et al. examined subcutaneous fat and there were no overlapping proteins with our results. In addition to differences in methods, the lack of common proteins could be due to the different locations from which the subcutaneous fat was obtained and/or different patient populations. In their study, Boden et al. [17] compared subcutaneous fat obtained from the upper thigh in six patients who were lean and had insulin sensitivity vs six patients with obesity and insulin resistance. Here, we compared subcutaneous fat obtained from the abdominal region of the trunk in 20 diabetic vs 22 nondiabetic patients, who all had obesity. There were also no common proteins in the study by Oliva et al. examining the omental proteome from patients with normal glucose tolerance vs gestational diabetes [23]. When our results were compared with the two remaining studies, apolipoprotein A-IV was differentially abundant in all proteome profiles in omentum from patients with type 2 diabetes [22,24]. Similar to our study, Kim et al. [24] found a decrease in abundance of apolipoprotein A-IV (−1.9 vs −2.2, respectively) while Murri et al. [22] found an increase (1.48). Additionally, annexin A1 was differentially abundant in our study and that of Murri et al. [22] (1.54 vs −2.35, respectively) and myosin 1c was decreased in abundance in our study and that of Kim et al. (−6.36 vs −5.9, respectively). These cross-comparisons highlight the importance of recognizing 1) tissue sub-type, 2) tissue location, and 3) patient population, as there appears to be a significant and interesting dynamic emerging for this recently recognized organ, adipose tissue, that will take additional and ongoing studies to uncover. In that context, it should be duly noted that this area of research, while extremely important, is very new with the majority of studies carried out within the last decade.

While outside the context of this study, it is also important to comment on the growing connections between obesity, type 2 diabetes, and cancer, including pancreatic cancer [48–51]. Notably, some of the most interesting proteins highlighted in this study that were differentially abundant based on diabetic status, such as decorin, moesin, and GRP78, have been implicated in cancer initiation, progression, and/or therapeutic resistance [52–54]. From this perspective, we plan to extend this work to include a more focused view on obesity and cancer, specifically the relationship between type 2 diabetes and pancreatic cancer.

Finally, the limitations of this study must be considered. First, of the 48 subjects, there was only one male among 47 females, so that the findings may not be extrapolated to males. Given the small sample number and clinical heterogeneity of patients, this was intentional in the study design to avoid differences due to sex. Females were selected because they represent approximately 86% of the patients undergoing bariatric surgery at our institution. Second, due to the low abundance of protein extracted for many of the samples, we were forced to use data obtained from single injections. However, we also analyzed an internal quality control sample obtained from a random omental specimen, which was run in between every 8–10 samples (n=9). These data were analyzed to assess instrumental reproducibility throughout the entire dataset. The results indicated a high significance of reproducibility with R values ranging from 0.957–0.981, and an average relative standard deviation (RSD) across natural-log (ln) normalized spectral counts (N-SC) of 27.6%. Therefore, we feel these data are highly robust despite having to use single injection results. Lastly, the proteomic dataset was generated from patients who were stratified by diabetic status, and there was a significant difference in the presence of hyperlipidemia between the two groups. To address whether the differential abundance in the proteome was due to diabetic status and not lipid metabolism, we specifically sought to, 1) select proteins for validation based on suspected or known role in diabetes and metabolism, 2) perform validation studies in specimens from patients without lipid dysmetabolism and from diabetic patients on single drug therapy for diabetes that was not insulin or a thiazolidinedione, and 3) compare our results with previously published datasets comparing patients with insulin resistance vs insulin sensitivity or in patients with frank type 2 diabetes. Thus, taken together, we are encouraged that future investigation of novel proteins identified here will be important in understanding the pathogenesis or pathophysiology of type 2 diabetes.

## Conclusions

Adipose tissue is now regarded as a dynamic organ that contributes to the pathophysiology of dysmetabolism, with specific fat depots displaying unique metabolic and biological characteristics [6–11,18,21]. Here, we identified 19 proteins from subcutaneous adipose tissue and 41 proteins from omentum that were differentially abundant in diabetic vs nondiabetic patients with morbid obesity. Consistent with visceral fat playing a major role in dysmetabolism, there were more proteins identified in the omentum and the differentially abundant proteins in omentum better represented biological processes known to contribute to cellular energy homeostasis and/or the pathogenesis of type 2 diabetes. Two proteins, decorin and GRP78, were validated. Previously published data do implicate these proteins in the pathophysiology of type 2 diabetes. In addition, three proteins identified here have been identified as being differentially abundant in previously published studies of the omental proteome in diabetic vs nondiabetic patients [22–24]. Finally, while beyond the scope of this work, proteins highlighted in this study have known associations with cancer in general and pancreatic cancer specifically [52–54]. This is provocative since pancreatic cancer has been associated with both obesity and type 2 diabetes [48–51]. It is hoped that further investigation of the proteins and associated networks reported here will lead to novel therapeutic interventions in the treatment of type 2 diabetes mellitus.

## Figures and Tables

**Table 1:** Demographic and clinical characteristics^a^.

	All (n=42)^b^	Non-Diabetes (n=22)	Diabetes (n=20)	p value
Age (years)	42.60 ± 10.04	42.64 ± 10.46	42.55 ± 9.83	0.978
Sex (female)	42 (100%)	22 (100%)	20 (100%)	0.095
Body mass index (kg/m^2^)	52.14 ± 8.88	53.30 ± 8.04	50.79 ± 9.81	0.375
Hypertension	28 (66.7%)	12 (54.5%)	16 (80%)	0.081
Hyperlipidemia	11 (26.2%)	3 (13.6%)	8 (40%)	0.052
Fasting glucose (mg/dl)^c^	117.45 ± 37.44	105.71 ± 22.30	130.42 ± 46.33	0.035*
Total cholesterol (mg/dl)^d^	189.09 ± 42.74	178.81 ± 36.69	198.76 ± 46.76	0.184
Triglycerides (mg/dl)^d^	132.27 ± 60.25	128.38 ± 57.82	135.94 ± 64.00	0.725
LDL cholesterol (mg/dl)^e^	122.26 ± 34.41	113.75 ± 19.16	129.76 ± 42.95	0.194
HDL cholesterol (mg/dl)^e^	46.91 ± 10.98	48.53 ± 13.68	45.47 ± 8.09	0.440

a Data presented as median ± standard error or number (%)

b n, number

c All = 40, non-diabetes = 19 and diabetes = 21

d All = 33, nondiabetes = 16 and diabetes = 17

e All = 32, nondiabetes = 15 and diabetes = 17

* p value < 0.05 is significant

**Table 2:** Differentially abundant proteins in subcutaneous fat in diabetic vs nondiabetic patients.

Sequence ID	Sequence Name	Total Peptides	Fold Change	p-value*
Q8N823	Zinc finger protein 611	4	23.65	0.008
P48061	Stromal cell-derived factor 1	2	4.09	0.026
Q6PRD1	Probable G-protein coupled receptor 179	5	3.52	0.007
Q9BRD0	BUD13 homolog	2	3.48	0.014
P04271	Protein S100-B	2	2.83	0.038
P11021	78 kDa glucose-regulated protein	7	2.55	0.007
P06748	Nucleophosmin	3	2.45	0.010
Q9BZE4	Nucleolar GTP-binding protein 1	3	2.26	0.026
P35579	Myosin-9	26	2.11	0.036
P26038	Moesin	14	1.93	0.049
P09486	SPARC	9	1.74	0.014
P62805	Histone H4	2	1.60	0.024
P22352	Glutathione peroxidase 3	6	1.57	0.046
O14558	Heat shock protein beta-6	8	−1.63	0.040
O75128	Protein cordon-bleu	4	−1.65	0.010
Q9P2J8	Zinc finger protein 624	3	−1.75	0.049
Q9C0G0	Zinc finger protein 407	4	−1.87	0.042
P31949	Protein S100-A11	6	−3.51	0.024
P14854	Cytochrome c oxidase subunit 6B1	4	−4.03	0.001

* p value < 0.05 is significant

**Table 3:** Differentially abundant proteins in omentum in diabetic vs nondiabetic patients

Sequence ID	Sequence Name	Total Peptides	Fold Change	p value*
P07585	Decorin	12	11.73	0.004
Q15652	Probable JmjC domain-containing histone demethylation protein 2C	4	9.17	0.001
P11021	78 kDa glucose-regulated protein	7	7.09	0.002
Q9P2D1	Chromodomain-helicase-DNA-binding protein 7	3	6.60	0.005
O95416	Transcription factor SOX-14	3	6.60	0.008
Q8N823	Zinc finger protein 611	4	6.00	0.003
P08620	Fibroblast growth factor 4	2	4.03	0.005
Q92736	Ryanodine receptor 2	3	3.85	0.007
P00450	Ceruloplasmin	34	2.65	0.016
P02585	Troponin C, skeletal muscle	3	2.48	0.008
P02760	Protein AMBP	8	2.08	0.007
P26038	Moesin	14	2.03	0.033
Q9UNA0	A disintegrin and metalloproteinase with thrombospondin motifs 5	2	1.73	0.024
P02787	Serotransferrin	61	1.72	0.023
P04083	Annexin A1	12	1.54	0.046
P06454	Prothymosin alpha	6	−1.64	0.024
Q68DD2	Cytosolic phospholipase A2 zeta	2	−1.79	0.007
O76070	Gamma-synuclein	11	−1.83	0.038
P06727	Apolipoprotein A-IV	17	−1.90	0.048
P43652	Afamin	18	−1.94	0.027
Q02952	A-kinase anchor protein 12	14	−1.96	0.003
Q05682	Caldesmon	17	−1.96	0.015
O75128	Protein cordon-bleu	4	−2.07	0.018
P61604	10 kDa heat shock protein, mitochondrial	6	−2.27	0.049
P18859	ATP synthase-coupling factor 6, mitochondrial	3	−2.44	0.041
O95810	Serum deprivation-response protein	12	−2.50	0.001
Q15121	Astrocytic phosphoprotein PEA-15	6	−2.55	0.023
P27816	Microtubule-associated protein 4	15	−2.66	0.010
B9ZVR1	Microtubule-associated protein	9	−2.68	0.007
Q0VG54	TNS1 protein	11	−2.68	0.000
P14209	CD99 antigen	4	−2.73	0.016
P10321	HLA class I histocompatibility antigen, Cw-7 alpha chain	2	−2.73	0.025
Q9BZ29	Dedicator of cytokinesis protein 9	4	−2.86	0.003
Q05639	Elongation factor 1-alpha 2	5	−2.91	0.033
P98171	Rho GTPase-activating protein 4	3	−2.99	0.031
Q9UFH2	Dynein heavy chain 17, axonemal	2	−3.01	0.001
B8ZWD2	Diazepam binding inhibitor (GABA receptor modulator, acyl-Coenzyme A binding protein), isoform CRA_a	11	−3.53	0.000
P48681	Nestin	4	−4.55	0.000
B7ZM78	Girdin (CCDC88A protein)	3	−4.85	0.013
O00159	Unconventional myosin-Ic	5	−6.36	0.000
P14854	Cytochrome c oxidase subunit 6B1	4	−7.27	0.013

* p value < 0.05 is significant

**Table 4:** Differentially abundant proteins in diabetic and nondiabetic patients by biological and metabolic processes.

	Sc	Om
Actin cytoskeleton	Protein S100-A11 (−3.51)	Unconventional myosin-1c (−6.36)
	**Protein cordon-bleu (−1.65)**^a^	Girdin (−4.85)
	**Moesin (1.93)**	Rho GTPase-activating protein 4 (−2.99)
		Elongation factor 1-alpha 2 (−2.91)
	Myosin-9 (2.11)	CD99 antigen (−2.73)
		TNS 1 protein (−2.68)
		Serum deprivation-response protein (2.50)
		**Protein cordon-bleu (−2.07)**
		A-kinase anchor protein 12 (−1.96)
		Caldesmon (−1.96)
		Annexin A1 (1.54)
		**Moesin (2.03)**

Lipid metabolism	**Moesin (1.93)**	Diazepam binding inhibitor (−3.53)
		Apolipoprotein A-IV (−1.90)
		Gamma-synuclein (−1.83)
		Annexin A1 (1.54)
		Serotransferrin (1.72)
		**Moesin (2.03)**
		Ceruloplasmin (2.65)

Oxidative stress	Protein S100-B (2.83)	Afamin (−1.94)
		Apolipoprotein A-IV (−1.90)
		Serotransferrin (1.72)
		Ceruloplasmin (2.65)

Cell signaling		Girdin (−4.85)
		A-kinase anchor protein 12 (−1.96)
		Decorin (11.73)

Mitochondrial function	**Cytochrome c oxidase subunit 6B1** **(−4.03)**	**Cytochrome c oxidase subunit 6B1** **(−7.27)**
	Stromal cell-derived factor 1 (4.09)	10 kDa heat shock protein (−2.72)
		ATP synthase-coupling factor 6 (−2.44)
		Ryanodine receptor 2 (3.85)

ER stress	**78 kDa glucose- regulated protein (2.55)**	**78 kDa glucose-regulated protein (7.09)**

a Proteins in bold were differentially abundant in both subcutaneous fat (Sc) and omentum (Om).

**Table 5:** Comparison of cited literature with current study.

Study	Fat depot	Patient population	Common proteins with current study	Fold change (cited vs current study)
Boden *et al*^17^	Subcutaneous fat	Lean, insulin sensitive vs obese, insulin resistant	None	
Oliva *et al*^23^	Omentum	Normal glucose tolerance vs gestational diabetes	None	
Murri *et al*^22^	Omentum	Pre-obese, diabetes vs pre-obese, normal glucose tolerance	Apolipoprotein A-IV Annexin A1	1.48 vs −1.9 −2.35 vs 1.54
Kim *et al*^24^	Omentum	Lean, normal glucose tolerance vs lean, early diabetes	Apolipoprotein A-IV Myosin 1c	−2.2 vs −1.9 −5.9 vs −6.36

## Abbreviations

ATF6: Activating Transcription Factor 6
GRP78: Glucose-Regulated Protein
IPA: Ingenuity Pathway Analysis
IRE1: Inositol-Requiring Enzyme 1
PERK: PKR-like ER Kinase
